# Altered regional homogeneity in experimentally induced low back pain: a resting-state fMRI study

**DOI:** 10.1186/1743-0003-11-115

**Published:** 2014-07-31

**Authors:** Shan-shan Zhang, Wen Wu, Zi-ping Liu, Guo-zhi Huang, Shi-gui Guo, Jian-ming Yang

**Affiliations:** 1 Department of Rehabilitation Medicine, Zhujiang Hospital, Southern Medical University, Guangzhou 510282, People's Republic of China; 2 Department of Radiology, Zhujiang Hospital, Southern Medical University, Guangzhou 510282, People's Republic of China

**Keywords:** Low back pain, Functional magnetic resonance imaging, Resting state, Regional homogeneity

## Abstract

**Background:**

Functional imaging studies have indicated that patients with low back pain can have significant reductions in cerebral cortex grey matter. However, the mechanisms governing the nociceptive pathways in the human brain are unclear. The aim of this study was to use functional magnetic resonance imaging (fMRI) and regional homogeneity (ReHo) to investigate changes in resting-state brain activity in subjects that experienced experimentally induced low back pain.

**Methods:**

Healthy subjects (n = 15) underwent fMRI (3.0 T) at baseline and during painful stimulation (intramuscular injection of 3% hypertonic saline).

**Results:**

Compared to the scans conducted at baseline, scans conducted during experimentally induced low back pain showed increased ReHo on the right side in the medial prefrontal cortex, precuneus, insula, parahippocampal gyrus and cerebellum (posterior lobe), but decreased ReHo in the primary somatosensory cortex, anterior cingulate cortex and parahippocampal gyrus on the left side. The right inferior parietal lobule also showed a decreased ReHo (*P* < 0.05, cluster threshold ≥10).

**Conclusions:**

These findings suggest that abnormally spontaneous resting-state activity in some brain regions may be associated with pain processing. These changes in neural activity may contribute to the recognition, execution, memory and emotional processing of acute low back pain.

## Introduction

Pain is processed in multiple brain areas, thus indicating the complexity of pain perception. Chronic low back pain (LBP) is increasingly viewed as a condition that affects normal brain function beyond the neural activity related to pain perception, leads to mental health problems, including depression, anxiety and sleeping disturbances [1,2]. Irrespective of the location, nature or course of the different pain syndromes, our understanding of the neural mechanisms of LBP has progressed through investigations examining the structural changes, relating marked reductions of grey matter in the medial prefrontal cortex (mPFC), anterior cingulate cortex (ACC) and insular cortices [3,4]. These studies have shown that grey matter changes are correlated with not only cortical activity but also the duration,and intensity of pain [5], suggesting that the structural changes in the brain result from functional changes. In the past decade, neuroimaging techniques have been used to identify functional changes that are important for cortical processing of pain. Individuals experiencing pain show abnormal neural activity in response to nociceptive stimuli [1,6-8]. Functional magnetic resonance imaging (fMRI) studies examining chronic LBP have indicated that the temporal and spatial properties of functional connectivity are disrupted when pain is perceived [2,9,10]. Moreover, chronic LBP can induce negative affective responses, such as unpleasantness, suffering, and other negative effects, which induce a pronounced activation of the pain matrix [11].

Although changes in neural activity related to chronic LBP have been extensively studied, little is known about how a network of selective painful stimuli is spatially represented in acute condition. Acute pain is typically accompanied by brief intervals of unpleasant emotions, anxiety, fear or avoidance behaviour. We suspect that brain activation patterns differ between individuals with chronic pain and acute pain. Considering acute LBP symptoms are paroxysmal and it is difficult for subjects to endure painful stimuli during fMRI scanning that can last more than 5 min, a proper pain model should be established in healthy subjects to improve the understanding of the neural mechanisms related underlying acute LBP. Intramuscular hypertonic saline injection is considered as a valid experimental model of acute pain in humans, and has been used in studies investigating orofacial muscle pain [12], dynamic neural activation [13] and discrete changes in cortical activity [14]. The advantage of this method is that it directly stimulates the muscle, thus causing persistent nociceptive stimulation that would enable a mechanistic study of brain activity during pain perception. Investigating brain activity during acute LBP would also help to explore the potential mechanisms through which acute pain can progress to chronic pain.

Neuroimaging can identify subtle changes in brain networks, and therefore can detect and localise nociceptive responses in the brain [15,16]. Resting-state fMRI has revealed that spontaneous neural activity is highly correlated with low frequency (<0.08 Hz) blood-oxygen-level-dependent signals [17,18]. Other studies suggest that brain fMRI can reveal the biochemical, structural and functional information that reflects physiological integrity in vivo [19]. Basic sensory and motor networks as well as several higher-order resting-state networks (RSNs) that primarily contain interconnections between heteromodal associated cortical regions can be identified by fMRI [20]. The default mode network (DMN), one of several RSNs, is the most thoroughly investigated and stable RSNs that provides ‘a balance of opposing forces’ and enhances ‘the maintenance of information for interpreting, responding to and even predicting environmental demands’ [18]. Acute muscle pain can alter brain dynamics, particularly responses of the DMN [14,21,22]. Moreover, somatotopic reorganisation occurs in the primary and secondary sensory areas in response to both chronic and experimentally induced acute pain [23-25].

Here, we present a novel method based on regional homogeneity (ReHo) to analyse spontaneous resting-state brain activity. The ReHo quantifies the similarities among several time series of fMRI signals and takes into account the resting-state activity across the entire brain. Compared to classic model-driven methods, ReHo appears more sensitive to spontaneous haemodynamic responses and can detect unpredictable haemodynamic responses that model-driven methods fail to identify. Because ReHo represents the synchronised neural activity in a specific region, it can serve as a complementary tool for investigating resting brain activity and regional stability [26]. Recent studies have demonstrated that abnormal ReHo can occur in migraine [27], Alzheimer’s disease [28] and Parkinson’s disease [29]. These results confirm the diagnostic validity of ReHo for neuropsychiatric disorders. However, it remains unclear whether subjects with acute LBP demonstrate changes in regional coherence in response to noxious stimuli.

Based on previous evidence showing extensively altered brain dynamics in chronic low back pain patients [2,8], we report the generality of the results presented by Apkarian [1] by determining whether dynamic changes in RSN with spontaneous brain activity occur with tonic muscle pain. We hypothesised that changes in ReHo measurements of brain activity should be evident in experimentally induced acute LBP. The expected altered brain processing should involve mainly the brain structures mediating the recognition and emotional dimensions of pain, including the DMN, insula and hippocampus cortex.

## Materials and methods

### Participating subjects

Healthy subjects (n = 15) similar in age (22–30 years old, mean = 25.7), education (14–21 years, mean = 17) and approximately equal numbers’ gender (eight males and seven females) participated in this study. All subjects were right-handed and all gave informed consent to procedures approved by the Institutional Review Board (IRB) of Zhujiang Hospital Affiliated to Southern Medical University (Guangzhou). Inclusion criteria included: (1) Body mass index (BMI) with a range of ± 10% from standard BMI (Mainland, China); (2) No neurological or psychiatric illnesses (i.e. multiple sclerosis, epilepsy); (3) No pain (including dysmenorrhea) or drug use (i.e. antipyretics, sleeping pills) within the last month; (4) Self-rating anxiety scale (SAS) [30] and self-rating depression scale (SDS) [31] scores < 50.

### Stimulus and data acquisitions

With subjects in a prone position, a fine plastic cannula (24 gauge) attached to a 1 ml syringe containing sterile hypertonic (3%) saline was inserted 2 cm deep into the right back muscle, at the level of lumbar vertebra 4, prior to scanning for 10 min. The pain that caused by the needle subsided to pre-injection levels within approximately 20 s under non-anesthesia condition. A 3 T Philips Achieva whole-body scanner with single shot gradient echo-planar imaging (GRE-EPI) capability was used to conduct fMRI scans using a standard radio-frequency head coil. Multislice T_2_*-weighted scans were obtained using the following parameters: repetition time (TR) = 3 s; echo time (TE) = 40 ms; flip angle = 90°; field of view (FOV) = 220 mm × 220 mm; matrix = 64 × 64; 3.54 mm × 3.54 mm in-plane resolution; slice thickness, 5 mm; slice gap, 0.5 mm; 24 axial slices. Each subject underwent two identical fMRI at health status as baseline and after hypertonic saline as the pain stimulus. Following a 318 s baseline, all 15 subjects received an intramuscular injection of 3% hypertonic saline (0.3 ml) and the same fMRI scans were repeated 20 s after the injection. A high resolution T_1_-weighted fast spin echo anatomical scan was also acquired with TR/TE = 500/14 ms; matrix = 256 × 256; and FOV of 220 mm before baseline. Subjects were asked to remain still in the scanner and to keep their mind blank, eyes closed and avoid falling asleep. While still inside the scanner after scans, subjects were asked to provide verbal responses on their pain intensity using a visual analogue scale (VAS) [32], and a homemade mood scale for pain unpleasantness (including distressing, horrible). Moreover, subjects were required to rate the maximal pain intensity (0 ~ 10, 0 = “no pain” and 10 = “worst pain imaginable”) and unpleasantness (0 ~ 10, 0 = “Minimum” and 10 = “Maximal”), which evaluated the difference among the test before, on and after, as well as describe the time of onset, peak and cessation of pain. The locations of significantly activated clusters in the brain were determined by using standard anatomical landmarks (e.g. the postcentral gyrus is defined as S1).

### ReHo analysis

The BOLD signal was preprocessed using Data Processing Assistant for Resting-State fMRI (DPARSF, http://www.restfmri.net/forum/DPARSF) including image conversion, removal of the first ten volumes, slice-timing correction, motion correction, intensity normalization, detrending and filtering. All subjects’ head movements were less than 2 mm in translation and 2° in rotation by obtaining motion time courses (no subjects were excluded). Each individual’s functional images were normalized to the standard EPI templates and resampled with 3 × 3 × 3 mm^3^ resolution. A linear high-pass consisting of edge and high frequency artifact removal by linear regression was performed. A zero lag finite impulse response filter was applied to the band pass filter (0.01 Hz < f < 0.08Hz). The output data was then processed to produce ReHo map image files after filtering. The ReHo analysis was based on previous reports [26] and calculated as Kendall’s coefficient to measure the ReHo or similarity of ranked time series of a given voxel with its nearest 26 neighboring voxels in a voxel-wise way. The Kendall’s coefficient value was calculated to this voxel and an individual Kendall’s coefficient map was obtained for each subject. Each ReHo map was divided by its own mean ReHo within the mask for standardization purposes [26]. Spatial smoothing was conducted using a Gaussian kernel of full-width-half-maximum 6 mm to reduce the noise and residual differences in the gyral anatomy.

### Statistical analysis

Statistical analysis was performed using SPSS 13.0 software. Descriptive statistics (mean and standard deviation) were calculated for age, gender, education, pain intensity and pain unpleasantness. A one-way repeated measures analysis of variance (ANOVA) was used to compare the unpleasantness ratings before, during and after the MRI scans. Second level random effect analyses were used to create within group Statistical Parametric Mapping (SPM8, http://www.fil.ion.ucl.ac.uk/spm) and Xjview (http://www.alivelearn.net/xjview) for each network and to explore the ReHo differences between the two conditions. One-sample t-tests were performed for each network that had a threshold at the whole-brain cluster-level for *P* < 0.05 after correction for multiple comparisons using the false discovery rate (FDR) and a minimum cluster size of 10 voxels to extract the ReHo results across the subjects within each condition. To compare ReHo changes of the brain, two-tailed paired t-tests were used to compare group’s average percentage signal-intensity changes that threshold at the whole-brain cluster-level corrected at a significance level of 0.05 for multiple comparisons (FDR) using a minimum cluster size of 10 vcxels.

## Results

All patients were awake throughout the fMRI scans, rated the pain intensity as 3 or higher and did not produce head movements greater than 2 mm in translation or 2° in rotation. Therefore, data for all patients was included in the study.

### Pain perception

Unilateral (right) injections of hypertonic saline into the back muscle caused pain that began within 15 s of the injection and subsided to pre-injection levels within 10 min of the injection. The muscle pain was described as dull and aching with mild numbness. The average maximum pain score following the intramuscular injection was 6.33 ± 2.29 (range, 3–9). As the muscle pain was induced, subjects described pain that rostrally progressed and encompassed a region surrounding the injection site with a 2–3 cm diameter. During the test, ‘distressing’ (2.92 ± 1.78) and ‘horrible’ (1.50 ± 1.62) scores, which indicated that subjects experienced mild distress or dread. However, the differences of pain unpleasantness before, during or after the MRI scans were not statistically significant (ANOVA, *P >* 0.05).

### fMRI ReHo alterations

**Figure 1:**
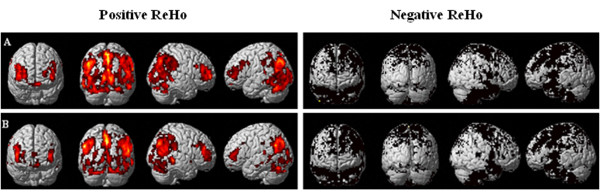
**Resting-state regional homogeneity (ReHo) of one-sample *t*-test in baseline (A) and pain status (B) (*P*** **< 0.05, cluster threshold ≥ 10).** Based on the normal topography of the selected resting-state networks, back muscle pain evoked ReHo abnormalities in the frontal lobe, parietal lobule, cingulate cortex and cerebellum. Note: Regional positive in ReHo for each network are shown as red, whereas negative are shown in black.

**Figure 2:**
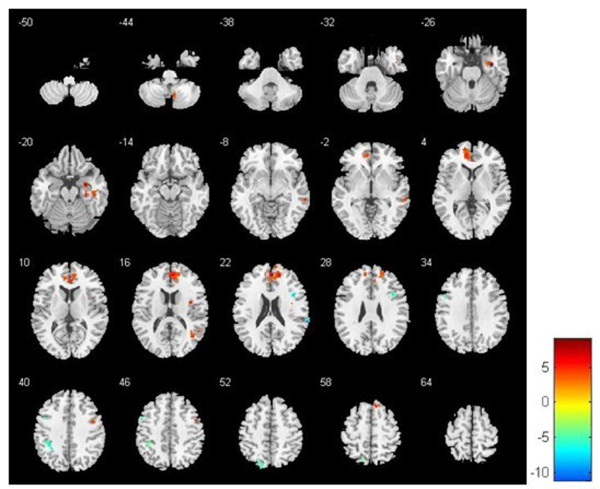
**Resting-state regional homogeneity (ReHo) of paired *t*-test between baseline and pain status (*P*** **< 0.05, cluster threshold ≥ 10).** Compared to baseline, acute low back pain demonstrated higher ReHo in the right medial prefrontal cortex, right middle frontal gyrus, right insula, right precuneus, right parahippocampal gyrus and right cerebellar tonsil. In contrast, pain status demonstrated lower ReHo in the right superior temporal gyrus, left middle temporal gyrus, left primary somatosensory cortex (S1), left anterior cingulate cortex (ACC), left parahippocampal gyrus and right inferior parietal lobule. Note: Status-averaged increases are red–yellow and decreases are blue–green.

The ReHo maps for pain status and baseline were consistent (Figure 1), but there were significant differences in brain activity (*P* < 0.05, FDR, cluster threshold ≥10). Second level analysis revealed several differences among the frontal lobe, temporal lobe, parietal lobe and cingulate cortex. Increased pain status scores corresponded to increased ReHo in the right mPFC, right middle frontal gyrus, right insula, right precuneus, right parahippocampal gyrus and right cerebellum (posterior lobe-cerebellar tonsil; Table 1). In contrast, the pain status scores corresponded to significant decreases in ReHo values in the right superior temporal gyrus, left middle temporal gyrus, left primary somatosensory cortex (S1), left ACC, left parahippocampal gyrus and right inferior parietal lobule (IPL; Figure 2). These changes suggest the spatial topography of the brain was altered with experimentally induced LBP.

**Table 1:** Resting-state regional homogeneity alterations corresponding to pain status

**Brain region**	**BA**	**Cluster sizes**	**t-value**	**P value**	**Peak MNI coodinate**		
	**(voxels)**		**(Corr.)**		**X**	**Y**	**Z**
R medial prefrontal cortex	8	12	6.02	<.05	9	27	57
R middle frontal gyrus	9	211	10.87	<.05	3	48	21
L middle temporal gyrus	21	14	−5.49	<.05	−66	−54	3
R superior temporal gyrus	38	12	−5.59	<.05	54	12	−18
L primary somatosensory cortex	2	19	−3.88	<.05	−60	−21	42
R inferior parietal lobule	40	10	−10.94	<.05	66	−36	21
L parahippocampal gyrus	―	11	−5.62	<.05	−9	−3	−21
R parahippocampal gyrus	35	29	9.51	<.05	30	−6	−21
L anterior cingulate cortex	32	12	−4.74	<.05	−6	21	39
R precuneus	7	14	4.70	<.05	18	−66	33
R insula	13	10	5.47	<.05	39	0	18
R cerebellar tonsil	―	13	5.30	<.05	9	−60	−48

## Discussion

In the present experiment, we used the ReHo method to investigate changes in experimentally induced LBP and examined pain processing among several brain regions. This investigation was motivated by hypothesis that persistent pain can induce changes in the brain beyond those that take place in the cortical circuits typically involved in pain processing. Our findings confirm this hypothesis and demonstrate that acute LBP corresponds to severe ReHo changes in several brain regions. Thus, using back pain to infer relevant information about pain processing in the brain with LBP is fully justified. Our approach was prolific and revealed that acute LBP affects normal brain function in the resting state.

Back pain evoked abnormal ReHo in the DMN. The DMN is characterised by balanced positive and negative correlations between the activity of the mPFC, PCC/precuneus, IPL and mesial temporal lobe structures. Furthermore, this activity correlates with behaviour, emotional measures and self-referential processes [33-36]. Consistent with previous findings where acute painful stimuli disrupted DMN activity [14,21,22], the present findings show abnormally spontaneous resting-state activity in DMN in acute LBP. The mPFC and precuneus exhibit significantly higher ReHo values than other brain regions. The mPFC is associated with processing emotional information and mediating functional interactions among brain regions that participate in pain processing [36]; the precuneus is likely involved in shifting attention between different spatial locations [37]. Therefore, changes in ReHo may reflect pain that is accompanied by the processing of emotionally intense information. Moreover, increased pain levels corresponded to decreased ReHo in the middle temporal gyrus. This may reflect abnormalities in experience-dependent maturation of cortical functional specialisation, and could be the consequence of increased inhibitory drive or asynchronous neuronal firing.

Some imaging studies with humans reported that muscle pain evoked signal increases in the mPFC, insula, S1 and cerebellar cortices [22,38,39]. Our study showed that pain perceived in back muscle evoked higher ReHo values in the mPFC, insula and cerebellum that were accompanied by lower values in the left S1 and right IPL. Considering the lateral pathway projects to S1, this cortical area is considered to encode the sensory-discriminative aspects of pain, such as location, intensity and quality [40,41]. The difference in results between the present and previous investigations may have resulted from differences in how noxious stimuli were delivered. We used stimuli that evoked pain in areas that were relatively larger than those in previous studies that delivered stimuli with heat thermodes or lasers. Furthermore, the changes in ReHo for S1 and contralateral IPL may be related to the earlier observation of somatotopic changes associated with pain intensity, and explain the sensory disturbances common to LBP.

A previous study reported an increased ReHo in the left ACC with an increased chronic back pain [10]. This is inconsistent with our present finding that ReHo decreased with acute LBP that prompted different ACC activation patterns between individuals with persistent pain and healthy subjects [42]. ACC participates in pain perception and integrating the sensory, attentional and cognitive components of pain [43,44]. The ReHo decreases in ACC suggests reductions in efficient pain processing or compensatory damage in functionally relevant regions, such as the prefrontal cortex and caudate [45]. It is well documented that pain may interrupt cognition and sustained attention to direct action toward a painful stimulus or threat [19]. The data further support the idea that cingulotomy alters pain perception so that pain no longer interferes with an individual’s daily activities [46].

Interestingly, we observed that activity evoked with acute painful stimulation was significantly different among brain regions. ReHo values particularly decreased in the left parahippocampal gyrus but increased in the right parahippocampal gyrus, as compared with the ‘no pain’ condition. The parahippocampal cortex provides the major poly-sensory input to the hippocampus and receives many combinations of sensory information [47]. In particular, the right parahippocampal gyrus is more involved in detecting and recognising pain than just perceiving pain [48]. The opposite results during acute muscle pain stimulation support the idea that left and right parahippocampal regions play different roles in attenuating pain perception. Therefore, abnormal ReHo results may represent the aberrant conditioning to sensory stimuli that occurs in LBP.

Our findings consequently not only indicate that regional resting-state abnormalities are associated with functional modulation during pain processing but also that changes evoked with acute LBP differ from those evoked with chronic LBP. Future studies using larger sample sizes with a sufficient number of subjects for performing meaningful comparisons between different gender disorders are needed. Furthermore, abundant phenotypic data is needed to help confirm these preliminary findings and to fully characterise changes in RSNs that process LBP.

## Conclusions

We demonstrated that experimentally induced LBP is associated with substantial spatial changes in regional homogeneity for brain regions active in the resting state. The disruptions of ReHo observed in the present study may be related to earlier observations of spontaneous brain activity occurring with persistent pain. We speculate that changes in ReHo are associated with the structural and functional plasticity of pain processing. These changes may account for the recognition, execution, emotional and memory processes involved in acute LBP. We hope that our findings will provide information that can help improve diagnostic and treatment strategies for patients with LBP.

## Competing interests

The authors declare that they have no competing interests.

## Authors’ contributions

SSZ designed and conducted the experiment, performed the analysis and wrote the manuscript. WW designed and conducted the experiment and was involved in revising the manuscript. ZPL conducted the experiment and performed the analysis. GZH made contribution to conception and design, interpretation of data, and revising the manuscript. JMY contributed to conception and design of the study. SGG conducted the experiment and made contribution for the interpretation of data. All authors read and approved the final manuscript.
